# Mortality Pattern of Hospitalized Children in a Tertiary Care Hospital of Kolkata

**DOI:** 10.4103/0970-0218.42062

**Published:** 2008-07

**Authors:** Rabindra Nath Roy, Saswati Nandy, Prabha Shrivastava, Arindam Chakraborty, Malay Dasgupta, Tapan Kumar Kundu

**Affiliations:** Department of Community Medicine, R.G. Kar Medical College and Hospital, Kolkata, West Bengal, India; 1 Department of Paediatric Medicine, R.G. Kar Medical College and Hospital, Kolkata, West Bengal, India

## Introduction

The causes of mortality are often poorly documented in developing countries. The Medical Records Department in a teaching hospital has a system of compilation and retention of records, yet the acquisition of meaningful statistics from these records for health care planning and review is lacking. Mortality data from hospitalized patients reflect the causes of major illnesses and care-seeking behaviour of the community as well as the standard of care being provided. Records of vital events like death constitute an important component of the Health Information System. Hospital-based death records provide information regarding the causes of deaths, case fatality rates, age and sex distribution, which are of great importance in planning health care services. A better understanding of childhood mortality could contribute to a more effective approach to saving these lives. A country needs sound epidemiological information to prioritize, plan and implement public health programmes. There is a paucity of information about direct causes of child mortality in developing countries.(1) This information also provides the basis for patient care and helps the administration in managing day-to-day hospital affairs. The present study was aimed at finding the causes of mortality of inpatients in the Paediatric Department admitted during 2005 and provides epidemiological information related to child mortality.

## Materials and Methods

A retrospective analysis was conducted with records of patients who died in the Paediatric Department of R.G. Kar Medical College and Hospital, Kolkata, over a 12-month period from 1 January to 31 December 2005. R.G. Kar Medical College and Hospital is a tertiary-level referral centre with a total of 90 beds in the Paediatric Department. Children under 12 years of age with illness requiring hospitalization are admitted to the Paediatric Department both from the outpatient and emergency departments. A paediatrician is available for consultation round-the-clock under the guidance of senior consultants. There is a neonatal care unit in the department. As there is no trauma care or burn unit in this department, all surgical paediatric cases are admitted to the surgery department. Children suspected to suffer from cholera or other infectious diseases are not admitted in this hospital; they are subsequently referred to infectious disease (ID) hospitals. For our purpose, data related to different variables such as age, sex, cause of mortality, etc. were retrieved from the registers maintained in the department. Data were analysed using frequency, proportion and *Z*-test.

## Results

A total of 3983 children comprising of 58% males and 42% females were admitted to the Paediatric Department during 2005. Out of the total admitted cases, 549 (13.78%) were neonates, in which 37.34% died for different reasons. A total of 667 (16.74% of admissions) post-neonatal infants were admitted during that period, of which 81 (12.14%) died. Admissions of the 1-4-year and 5-11-year age groups were 1288 (32.32%) and 1479 (37.13%), respectively, in which 4.11 and 3.65% of cases died, respectively. An overall 9.87% death was noted in cases admitted to the Paediatric Department.

The mean time interval between admissions and deaths was 72.3 h (approximately 3 days). Further analysis of distribution of admissions and death intervals revealed that 55.47% of deaths occurred within 24 h of admission. The intervals between admissions and deaths were 24-48 h, 49-120 h and more than 120 h in 13.99, 18.83 and 11.70 of deaths, respectively.

The risk of death was more or less similar between both sexes in all age groups the overall proportion of deaths in males and females was 9.87 and 9.86%, respectively.

The analysis of seasonal variation of mortality among hospitalized patients revealed no significant difference in admissions and deaths across different months. Septicaemia (31.29%) was the leading cause of death among all paediatric age groups [Table 1]. Meningitis, meningoencephalitis and acute respiratory infection (ARI) were responsible for 12.72 and 7.63% of paediatric deaths, respectively. Birth asphyxia (42.93%) was the commonest cause of neonatal deaths [Table 1].

**Table 1:** Distribution of causes of death in different age groups

Age group	Causes of deaths	Numbers	Percentage
Neonates	Birth asphyxia	88	42.93
	Septicaemia	77	37.56
	Prematurity	18	8.79
	Meningitis	6	2.92
	Cong. anomalies	3	1.46
	Miscellaneous causes	13	6.34
	Total	205	100.00
Post-neonatal infants	Septicaemia	28	34.57
	ARI	24	29.63
	Cong. heart disease	12	14.81
	Meningitis	10	12.35
	Miscellaneous causes	7	8.64
	Total	81	100.00
1-4 years children	Meningoencephalitis	16	30.19
	Septicaemia	14	26.42
	ARI	6	11.32
	Congenital abnormalities	2	3.77
	Acute lymphatic leukaemia	2	3.77
	Miscellaneous causes	13	24.53
	Total	53	100.00
5-11 years children	Meningoencephalitis	18	33.33
	Hepatic coma	6	11.11
	Septicaemia	4	7.41
	Poisoning	3	5.56
	Seizure disorders	2	3.70
	Cerebral malaria	2	3.70
	Congenital heart disease	2	3.70
	Acute lymphatic leukaemia	2	3.70
	Miscellaneous causes	15	27.79
	Total	54	100.00

## Discussion

The number of admissions was more in males (2310) than females (1673), which could be related to the biological vulnerability of males to infections or discrimination against female offspring. The male preponderance of admission has been documented in various studies.(2)

The overall mortality among patients was 9.87%, which was higher than that observed in the Emergency Department (2.7%) at PGIMER, Chandigarh. Higher mortality in the present study may be due to the large number of admissions in critical conditions. The lower proportion of deaths in PGIMER was due to the fact that a significant proportion of visits to the emergency department was trivial in nature.(2)

The risk of death in children is closely related to the environment they live in, the antenatal care their mothers receive and care after birth. The risk is highest during neonatal period.(3) In the present study, approximately 37.34% neonatal cases died as compared to 9.87% deaths of children of all age groups, indicating that the risk of death was highest in the neonatal period followed by deaths in the post-neonatal period (16.74%). Several other studies have revealed that risk of death is comparatively higher during neonatal and post-neonatal infancy.(2) A girl child in India is 30-50% more likely to die between her first and fifth birthday.(4) However, in the present study no significant difference in risk between two sexes was observed [Table 2].

**Table 2:** Age and sex distribution of paediatric deaths

Age group	No. of male admissions	No. of deaths	% of death	No. of female admissions	No. of deaths	% of death	Z (*P*-value)
Neonates	344	119	34.59	205	86	41.95	1.71 (0.043)
Post-neonatal infants	385	47	12.21	282	34	12.06	0.04 (0.48)
1-4 years	720	28	3.89	568	25	4.40	0.09 (0.46)
≥5 years	861	34	3.95	618	20	3.24	0.73 (0.23)
Total	2310	228	9.87	1673	165	9.86	0.02 (0.49)

The number of admissions was higher in the months of August to November and lowest in the months of April to July; however, the proportion of deaths to admissions was more or less similar across different months of the year, indicating the lack in seasonal variability of deaths (not shown in the table). However, Singhi *et al.* observed peak numbers of emergency admissions in the winter months, which they thought to be due to rise in ARI and asthma cases.(2)

More than 55% of paediatric deaths occurred within 24 h of admission, which could be attributed to delay in care-seeking and referral. The causes of death in children are determined by many factors such as environmental, behavioural and quality of care during illnesses.

The causes of mortality vary across different age groups. Birth asphyxia and septicaemia were, respectively, the two most common causes of deaths in 42.93% and 37.56% of all neonatal deaths. Singh noted from hospital-based data that bacterial sepsis was a major cause of neonatal mortality in India. It was responsible for one-fourth to half of neonatal deaths.(5) ARI is the leading cause of death in young children worldwide. In the present study, ARI was responsible for 29.63% of post-neonatal deaths and 11.32% of deaths in the 1-4-year age group.

### Limitation of the study

During the analysis of records, we observed that the data were incomplete or some entries were inadequate, which were excluded from our study.

## Conclusion

The pattern of mortality in different age groups suggests a need for more comprehensive improvement in antenatal and newborn care. The shorter time interval between admissions and deaths in majority of cases indicates a need for timely referral and safe transportation of cases.
